# Supplementary material for: Heat Capacities of l-Histidine, l-Phenylalanine, l-Proline, l-Tryptophan and l-Tyrosine
Source: Molecules. 2021 Jul 15;26(14):4298. doi: 10.3390/molecules26144298 (PMC8305567; doi:10.3390/molecules26144298)
Supplement: Supplementary file 1 [file molecules-26-04298-s001.zip › molecules-1284954-supplementary.pdf]

Supplementary Materials

Heat capacities of L-histidine, L-phenylalanine, L-proline, L-tryptophan, and L-tyrosine

Václav Pokorný[a], Vojtěch Štejfa[a], Jakub Havlín[b], Květoslav Růžička[a], Michal Fulem[a,]*

[a] Department of Physical Chemistry, University of Chemistry and Technology, Prague, Technická 5, CZ-166 28 Prague 6, Czech Republic

[b] Central Laboratories, University of Chemistry and Technology, Prague, Technická 5, CZ-166 28 Prague 6, Czech Republic

*E-mail: fulemm@vscht.cz

Supplementary materials contain:

1) DSC thermogram and apparent heat capacity for L-phenylalanine showing the phase transition form I to form Ih.
2) Experimental heat capacity data for L-histidine, L-phenylalanine, L-proline, L-tryptophan, and L-tyrosine measured using QuantumDesign PPMS, SETARAM μDSC IIIa and PerkinElmer DSC 8500.
3) Tabulated thermodynamic functions (heat capacity, entropy, enthalpy, Gibbs' energy) of L-histidine, L-phenylalanine, L-proline, L-tryptophan, and L-tyrosine.

1) DSC thermogram for L-phenylalanine

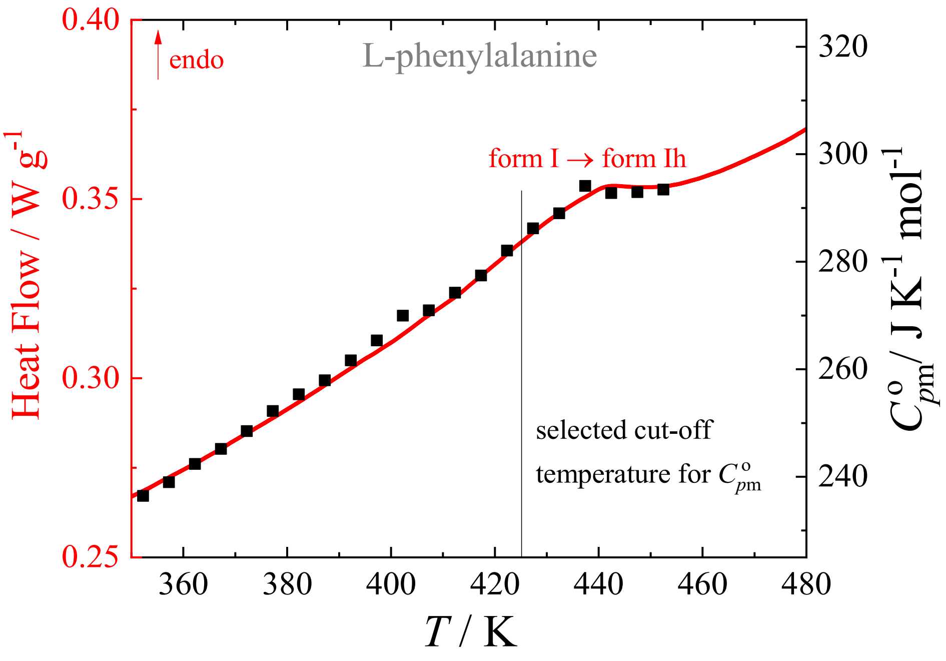

Figure S1. DSC thermogram (red line) and apparent high-temperature heat capacities C^{o}_{pm} (black symbols) for L-phenylalanine showing the phase transition form I to form Ih at around 440 K.

2) Experimental heat capacity data

Table S1. Experimental heat capacity of L-histidine (in J K^{-1} mol^{-1}) obtained using QuantumDesign PPMS.[a]

T / K	C^{o}_{pm}	δ_{rel} [b]/ %	T / K	C^{o}_{pm}	δ_{rel} [b]/ %	T / K	C^{o}_{pm}	δ_{rel} [b]/ %
303.029	181.55	-0.53	155.950	106.41	-0.02	35.100	24.070	-0.47
302.972	182.22	-0.15	155.946	106.39	-0.03	35.100	24.068	-0.48
295.787	178.80	0.09	148.954	102.77	-0.14	30.029	18.361	-0.12
295.994	178.65	-0.06	148.968	102.80	-0.11	30.030	18.383	0.00
288.797	175.83	0.54	141.967	99.046	-0.31	28.075	16.037	-0.65
288.978	175.61	0.36	141.965	99.177	-0.18	28.040	16.135	0.21
281.817	172.16	0.60	134.968	95.375	-0.39	26.025	13.878	0.36
281.975	171.75	0.31	134.941	95.574	-0.17	26.027	13.883	0.38
274.832	167.81	0.25	127.908	91.695	-0.37	24.026	11.681	0.32
274.982	167.55	0.04	127.845	91.922	-0.09	24.020	11.681	0.38
267.841	163.52	-0.09	120.922	87.990	-0.34	21.996	9.6109	0.81
267.991	163.58	-0.10	120.841	88.248	0.00	21.998	9.6097	0.77
260.856	159.78	-0.10	113.927	84.099	-0.40	19.950	7.6217	0.83
260.994	159.58	-0.27	113.846	84.254	-0.17	19.955	7.6293	0.87
253.858	156.03	-0.13	106.916	80.264	-0.25	17.978	5.8339	0.12
254.000	155.91	-0.25	106.858	80.378	-0.06	17.978	5.8057	-0.37
246.872	152.47	-0.04	100.888	76.905	-0.04	15.926	4.2070	-0.21
246.998	152.28	-0.21	100.832	76.975	0.10	15.957	4.2118	-0.63
239.871	148.93	0.05	95.843	74.115	0.31	13.911	2.8285	-0.93
239.989	148.77	-0.10	95.789	74.232	0.51	13.913	2.8291	-0.95
232.871	145.38	0.13	90.790	71.212	0.67	11.903	1.7498	-0.66
232.985	145.29	0.02	90.742	71.262	0.78	11.901	1.7540	-0.37
225.884	141.87	0.21	85.729	67.870	0.59	9.887	0.96960	0.76
225.987	141.73	0.08	85.681	67.986	0.81	9.894	0.96914	0.48
218.894	138.29	0.24	80.665	64.210	0.23	7.859	0.45910	0.75
218.988	138.25	0.17	80.613	64.285	0.41	7.866	0.45913	0.47
211.900	134.58	0.15	75.655	60.363	-0.28	6.339	0.23037	-0.17
211.991	134.36	-0.05	75.602	60.426	-0.11	6.290	0.22714	0.83
204.911	130.87	0.03	70.597	56.542	-0.45	5.242	0.12791	-0.56
204.994	130.73	-0.11	70.550	56.575	-0.33	5.242	0.12772	-0.70
197.921	127.28	-0.03	65.538	52.610	-0.50	4.362	0.07359	-0.42
197.993	127.19	-0.12	65.497	52.646	-0.37	4.361	0.07360	-0.34
190.931	123.89	0.06	60.491	48.652	-0.19	3.679	0.04390	-0.80
190.992	123.82	-0.02	60.463	48.540	-0.38	3.679	0.04388	-0.85
183.937	120.46	0.10	55.351	44.335	0.04	3.086	0.02586	-0.33
183.989	120.45	0.07	55.359	44.325	0.00	3.086	0.02584	-0.38
176.938	117.06	0.17	50.284	39.762	0.09	2.609	0.01571	1.43
176.985	117.08	0.16	50.284	39.768	0.11	2.609	0.01573	1.55
169.942	113.65	0.22	45.229	34.893	-0.02	2.222	0.00958	1.67
169.981	113.64	0.20	45.228	34.901	0.01	2.222	0.00958	1.62
162.946	110.05	0.12	40.169	29.625	-0.36	1.913	0.00580	-1.97
162.966	110.02	0.08	40.169	29.628	-0.35	1.912	0.00582	-1.40

[a] Standard uncertainty of temperature is $u(T)$=0.004 K, and the combined expanded uncertainty of heat capacity $U_c(C_{pm})$ with 0.95 level of confidence (k=2) is: $U_c(C_{pm})$=0.1 C_{pm} below 10 K; $U_c(C_{pm})$=0.03 C_{pm} in temperature range (10 to 40) K; $U_c(C_{pm})$=0.02 C_{pm} in temperature range (40 to 300) K. Values are reported with more digits than is justified by the experimental uncertainty to avoid round-off errors in calculations based on these results. Measurements are performed in vacuum (residual pressure $p<10^{-4}$ Pa).

[b] δ_{rel} is the percentage deviation of experimental heat capacity data from the smoothed values from Equations (1) and (2) using parameters listed in Table 5.

Table S2. Experimental heat capacity of L-histidine (in J K^{-1} mol^{-1}) at p = (100 ± 5) kPa obtained using SETARAM µDSC IIIa and PerkinElmer DSC 8500.

SETARAM µDSC IIIa[a]			PerkinElmer DSC 8500[b]		
m = 384.18 mg			m = 21.78 mg, SF = 0.976		
T / K	C^{o}_{pm}	δ_{rel} [c] / %	T / K	C^{o}_{pm}	δ_{rel} [c] /%
266.95	163.04	-0.09	307.32	184.64	-0.10
270.00	165.16	0.21	312.30	187.47	-0.01
275.00	167.72	0.14	317.29	190.18	0.01
280.00	170.04	-0.07	322.27	192.51	-0.15
285.00	172.45	-0.23	327.26	195.31	-0.07
290.00	175.09	-0.25	332.25	197.80	-0.15
295.00	177.85	-0.21	337.24	200.56	-0.08
300.00	180.47	-0.24	342.24	203.17	-0.08
305.00	183.23	-0.19	347.24	205.90	-0.02
310.00	185.98	-0.15	352.24	208.63	0.06
315.00	188.81	-0.06	357.24	210.91	-0.08
320.00	191.57	-0.01	362.24	213.39	-0.11
325.00	194.23	-0.01	367.24	215.95	-0.09
330.00	196.91	0.01	372.24	218.80	0.07
335.00	199.60	0.03	377.24	221.56	0.20
340.00	202.28	0.06	382.25	224.07	0.23
345.00	204.94	0.08	387.26	226.41	0.20
350.00	207.66	0.15	392.26	229.05	0.31
353.20	209.38	0.18	397.27	231.19	0.22
			402.29	232.82	-0.08
			407.30	234.72	-0.23
			412.32	237.26	-0.10
			417.33	239.52	-0.07
			422.35	241.60	-0.10
			427.37	243.72	-0.09
			432.39	245.72	-0.11
			437.41	248.30	0.12

[a] Standard uncertainty of temperature is $u(T)$ = 0.05 K, and the combined expanded uncertainty of the heat capacity is $U_c(C^{o}_{pm}) = 0.01\,C^{o}_{pm}$ (0.95 level of confidence).

[b] Standard uncertainty of temperature is $u(T)$ = 0.1 K, and the combined expanded uncertainty of the heat capacity is $U_c(C^{o}_{pm}) = 0.03\,C^{o}_{pm}$ (0.95 level of confidence). The raw experimental data were scaled by the *ad hoc* selected *SF* value to obtain superior agreement with the more accurate SETARAM µDSC IIIa results.

[c] δ_{rel} is the percentage deviation of experimental heat capacity data from the smoothed values from Equations (1) and (2) using parameters listed in Table 5.

Table S3. Experimental heat capacity of L-phenylalanine (in J K^{-1} mol^{-1}) at $p = (100 \pm 5)$ kPa.

SETARAM μDSC IIIa[a]			PerkinElmer DSC 8500[b]		
m = 472.14 mg			m = 16.98 mg, SF = 0.993		
T / K	C^{o}_{pm}	δ_{rel} [c] / %	T / K	C^{o}_{pm}	δ_{rel} [c] /%
262.19	178.54	-0.19	307.32	207.47	-0.07
265.00	180.61	-0.03	312.30	210.64	-0.08
270.00	183.94	0.07	317.29	214.29	0.13
275.00	187.15	0.09	322.27	217.02	-0.09
280.00	190.24	0.04	327.26	220.58	0.07
285.00	193.23	-0.06	332.25	223.36	-0.12
290.00	196.29	-0.12	337.24	226.24	-0.26
295.00	199.61	-0.06	342.24	229.56	-0.21
300.00	202.91	0.00	347.24	233.13	-0.05
305.00	206.16	0.02	352.24	236.43	-0.01
310.00	209.34	0.01	357.24	238.99	-0.28
315.00	212.65	0.05	362.24	242.37	-0.19
320.00	215.85	0.04	367.24	245.17	-0.35
325.00	219.02	0.02	372.24	248.48	-0.29
330.00	222.11	-0.03	377.24	252.23	-0.06
335.00	225.15	-0.11	382.25	255.34	-0.07
340.00	228.10	-0.22	387.26	257.92	-0.29
345.00	231.44	-0.16	392.26	261.65	-0.06
350.00	234.93	-0.04	397.27	265.34	0.16
355.00	237.99	-0.10	402.29	269.97	0.73
358.02	239.77	-0.16	407.30	270.96	-0.06
			412.32	274.24	0.02
			417.33	277.43	0.08
			422.35	282.08	0.65

[a] Standard uncertainty of temperature is $u(T)$ = 0.05 K, and the combined expanded uncertainty of the heat capacity is $U_c(C^{o}_{pm}) = 0.01\, C^{o}_{pm}$ (0.95 level of confidence).

[b] Standard uncertainty of temperature is $u(T)$ = 0.1 K, and the combined expanded uncertainty of the heat capacity is $U_c(C^{o}_{pm}) = 0.03\, C^{o}_{pm}$ (0.95 level of confidence). The raw experimental data were scaled by the *ad hoc* selected *SF* value to obtain superior agreement with the more accurate SETARAM μDSC IIIa results.

[c] δ_{rel} is the percentage deviation of experimental heat capacity data from the smoothed values from Equations (1) and (2) using parameters listed in Table 5.

Table S4. Experimental heat capacity of L-proline (in J K^{-1} mol^{-1}) at p = (100 ± 5) kPa.

SETARAM μDSC IIIa[a]			PerkinElmer DSC 8500[b]		
m = 429.09 mg			m = 14.71 mg, SF = 1.017		
T / K	C^{o}_{pm}	δ_{rel} [c] / %	T / K	C^{o}_{pm}	δ_{rel} [c] /%
262.31	133.42	-0.54	305.83	152.06	-1.02
265.00	134.85	-0.35	310.84	155.01	-0.61
270.00	137.20	-0.23	315.84	157.30	-0.64
275.00	139.44	-0.19	320.85	159.97	-0.45
280.00	141.54	-0.27	325.85	162.37	-0.45
285.00	143.61	-0.37	330.85	166.05	0.32
290.00	145.80	-0.40	335.85	168.35	0.22
295.00	148.19	-0.31	340.86	171.20	0.41
300.00	150.69	-0.16	345.87	173.36	0.19
305.00	153.08	-0.10	350.87	175.78	0.11
310.00	155.44	-0.08	355.88	178.33	0.08
315.00	157.85	-0.04	360.89	181.05	0.13
320.00	160.26	-0.01	365.89	183.78	0.20
325.00	162.67	-0.01	370.90	186.23	0.11
330.00	164.94	-0.10	375.90	188.89	0.15
335.00	167.25	-0.19	380.89	191.29	0.09
340.00	169.58	-0.28	385.89	193.82	0.12
345.00	172.02	-0.32	390.90	196.27	0.14
350.00	174.88	-0.15	395.90	198.36	0.04
355.00	177.50	-0.13	400.87	200.22	-0.12
358.02	178.99	-0.18	405.87	202.21	-0.16
			410.91	204.22	-0.12
			415.92	206.21	0.00
			420.93	207.71	-0.04
			425.93	209.00	-0.10
			430.92	210.53	0.07
			435.92	211.57	0.10

[a] Standard uncertainty of temperature is $u(T)$ = 0.05 K, and the combined expanded uncertainty of the heat capacity is $U_c(C^{o}_{pm}) = 0.01\, C^{o}_{pm}$ (0.95 level of confidence).

[b] Standard uncertainty of temperature is $u(T)$ = 0.1 K, and the combined expanded uncertainty of the heat capacity is $U_c(C^{o}_{pm}) = 0.03\, C^{o}_{pm}$ (0.95 level of confidence). The raw experimental data were scaled by the *ad hoc* selected *SF* value to obtain superior agreement with the more accurate SETARAM μDSC IIIa results.

[c] δ_{rel} is the percentage deviation of experimental heat capacity data from the smoothed values from Equations (1) and (2) using parameters listed in Table 5.

Table S5: Experimental heat capacity of L-tryptophan (in J K^{-1} mol^{-1}) at p = (100 ± 5) kPa.

SETARAM μDSC IIIa[a]			PerkinElmer DSC 8500[b]		
m = 391.53 mg			m = 20.84 mg, SF = 1.004		
T / K	C^{o}_{pm}	δ_{rel} [c] / %	T / K	C^{o}_{pm}	δ_{rel} [c] /%
266.17	212.12	-0.49	307.32	244.49	-0.03
270.00	215.82	-0.12	312.30	248.21	-0.05
275.00	220.25	0.16	317.29	252.45	0.14
280.00	224.13	0.18	322.27	255.65	-0.08
285.00	227.94	0.18	327.26	259.51	-0.03
290.00	231.68	0.14	332.25	262.66	-0.25
295.00	235.29	0.05	337.24	266.43	-0.22
300.00	238.90	-0.04	342.24	270.06	-0.24
305.00	242.56	-0.10	347.24	274.26	-0.04
310.00	246.24	-0.14	352.24	278.08	0.02
315.00	249.99	-0.15	357.24	280.87	-0.27
320.00	253.69	-0.18	362.24	284.69	-0.18
325.00	257.49	-0.16	367.24	287.70	-0.36
330.00	261.25	-0.15	372.24	291.84	-0.14
335.00	265.18	-0.07	377.24	296.28	0.19
340.00	269.01	-0.02	382.25	300.57	0.48
345.00	272.68	-0.02	387.26	303.84	0.44
350.00	276.52	0.05	392.26	307.12	0.42
353.33	279.12	0.11	397.27	309.98	0.28
			402.29	313.85	0.48
			407.30	314.44	-0.35
			412.32	318.14	-0.16
			417.33	321.83	0.04
			422.35	324.91	0.06
			427.37	325.66	-0.60
			432.39	330.12	-0.11
			437.41	333.96	0.20

[a] Standard uncertainty of temperature is $u(T)$ = 0.05 K, and the combined expanded uncertainty of the heat capacity is $U_c(C^{o}_{pm}) = 0.01\, C^{o}_{pm}$ (0.95 level of confidence).

[b] Standard uncertainty of temperature is $u(T)$ = 0.1 K, and the combined expanded uncertainty of the heat capacity is $U_c(C^{o}_{pm}) = 0.03\, C^{o}_{pm}$ (0.95 level of confidence). The raw experimental data were scaled by the *ad hoc* selected *SF* value to obtain superior agreement with the more accurate SETARAM μDSC IIIa results.

[c] δ_{rel} is the percentage deviation of experimental heat capacity data from the smoothed values from Equations (1) and (2) using parameters listed in Table 5

Table S6: Experimental heat capacity of L-tyrosine (in J K^{-1} mol^{-1}) at p = (100 ± 5) kPa

SETARAM μDSC IIIa[a]			PerkinElmer DSC 8500[b]		
m = 410.88 mg			m = 21.38 mg, SF = 1.001		
T / K	C^{o}_{pm}	δ_{rel} [c] / %	T / K	C^{o}_{pm}	δ_{rel} [c] /%
265.89	192.37	-0.54	307.32	221.18	-0.28
270.00	195.91	-0.16	312.30	224.63	-0.28
275.00	199.93	0.16	317.29	228.63	-0.04
280.00	203.43	0.20	322.27	231.71	-0.21
285.00	206.78	0.16	327.26	235.05	-0.27
290.00	210.10	0.11	332.25	238.86	-0.14
295.00	213.33	0.01	337.24	242.45	-0.10
300.00	216.59	-0.07	342.24	246.33	0.05
305.00	219.86	-0.15	347.24	249.98	0.10
310.00	223.22	-0.20	352.24	253.51	0.09
315.00	226.64	-0.21	357.24	256.41	-0.16
320.00	230.11	-0.22	362.24	260.18	-0.08
325.00	233.60	-0.21	367.24	263.47	-0.18
330.00	237.11	-0.21	372.24	266.93	-0.22
335.00	240.76	-0.14	377.24	271.11	0.00
340.00	244.37	-0.10	382.25	274.23	-0.17
345.00	248.03	-0.05	387.26	277.80	-0.18
350.00	251.79	0.04	392.26	282.15	0.09
353.36	254.45	0.15	397.27	285.81	0.10
			402.29	290.30	0.40
			407.30	292.69	-0.03
			412.32	296.35	-0.02
			417.33	300.57	0.17
			422.35	304.36	0.22
			427.37	308.32	0.32
			432.39	310.49	-0.16
			437.41	313.61	-0.33

[a] Standard uncertainty of temperature is $u(T)$ = 0.05 K, and the combined expanded uncertainty of the heat capacity is $U_c(C^{o}_{pm}) = 0.01\,C^{o}_{pm}$ (0.95 level of confidence).

[b] Standard uncertainty of temperature is $u(T)$ = 0.1 K, and the combined expanded uncertainty of the heat capacity is $U_c(C^{o}_{pm}) = 0.03\,C^{o}_{pm}$ (0.95 level of confidence). The raw experimental data were scaled by the *ad hoc* selected *SF* value to obtain superior agreement with the more accurate SETARAM μDSC IIIa results.

[c] δ_{rel} is the percentage deviation of experimental heat capacity data from the smoothed values from Equations (1) and (2) using parameters listed in Table 5

3) Tabulated thermodynamic functions

Standard thermodynamic functions were calculated using fundamental thermodynamic relationships (assuming residual entropy at 0 K for all crystalline amino acids to be 0 $J \cdot K^{-1} \cdot mol^{-1}$) and heat capacities $C^{\circ}_{p\mathrm{m}}(T)$ represented by Equations (1) and (2) using parameters listed in Table 5:

$$S^{\circ}_{\mathrm{m}}(T) = \int_0^T \frac{C^{\circ}_{p\mathrm{m}}(T)}{T} dT \qquad \text{(S1)}$$

$$\Delta_0^T H^{\circ}_{\mathrm{m}} = \int_0^T C^{\circ}_{p\mathrm{m}}(T)\, dT \qquad \text{(S2)}$$

$$\Delta_0^T G^{\circ}_{\mathrm{m}} = \Delta_0^T H^{\circ}_{\mathrm{m}} - TS^{\circ}_{\mathrm{m}}(T) \qquad \text{(S3)}$$

Table S7. Standard thermodynamic functions of L-histidine in the crystal state[a] at $p = 0.1$ MPa.[b]

T / K	C°_{pm} / J·K^{-1}·mol^{-1}	S°_{m} / J·K^{-1}·mol^{-1}	$\Delta_0^T H^{\circ}_{m}$ / kJ·mol^{-1}	$\Delta_0^T G^{\circ}_{m}$ / kJ·mol^{-1}
1[c]	7.816E-04	2.527E-04	1.908E-07	-6.194E-08
2	6.793E-03	2.179E-03	3.299E-06	-1.060E-06
3	2.379E-02	7.688E-03	1.745E-05	-5.616E-06
4	5.695E-02	1.863E-02	5.628E-05	-1.825E-05
5	1.115E-01	3.674E-02	1.384E-04	-4.527E-05
6	1.946E-01	6.391E-02	2.887E-04	-9.475E-05
7	3.151E-01	1.024E-01	5.400E-04	-1.769E-04
8	4.825E-01	1.548E-01	9.345E-04	-3.042E-04
9	7.072E-01	2.240E-01	1.524E-03	-4.921E-04
10	9.988E-01	3.130E-01	2.371E-03	-7.589E-04
11	1.365	4.248E-01	3.547E-03	-1.126E-03
12	1.808	5.620E-01	5.127E-03	-1.617E-03
13	2.324	7.266E-01	7.187E-03	-2.259E-03
14	2.910	9.198E-01	9.798E-03	-3.080E-03
15	3.560	1.142	1.303E-02	-4.108E-03
16	4.270	1.395	1.694E-02	-5.374E-03
17	5.033	1.676	2.159E-02	-6.907E-03
18	5.845	1.987	2.702E-02	-8.736E-03
19	6.703	2.325	3.329E-02	-1.089E-02
20	7.605	2.692	4.044E-02	-1.340E-02
25	12.70	4.920	9.085E-02	-3.216E-02
30	18.35	7.730	1.683E-01	-6.357E-02
35	24.07	10.99	2.744E-01	-1.102E-01
40	29.55	14.57	4.086E-01	-1.740E-01
45	34.67	18.35	5.694E-01	-2.562E-01
50	39.46	22.25	7.548E-01	-3.577E-01
55	44.01	26.23	9.636E-01	-4.788E-01
60	48.33	30.24	1.195	-6.200E-01
65	52.44	34.27	1.447	-7.813E-01
70	56.35	38.31	1.719	-9.627E-01
75	60.06	42.32	2.010	-1.164
80	63.60	46.31	2.319	-1.386
85	66.99	50.27	2.646	-1.627
90	70.24	54.19	2.989	-1.889
95	73.37	58.07	3.348	-2.169
100	76.40	61.91	3.722	-2.469
110	82.23	69.47	4.516	-3.126
120	87.79	76.87	5.366	-3.858
130	93.15	84.10	6.271	-4.663
140	98.35	91.20	7.228	-5.540
150	103.4	98.16	8.237	-6.487
160	108.5	105.0	9.297	-7.502
170	113.4	111.7	10.41	-8.586
180	118.4	118.3	11.57	-9.736
190	123.4	124.9	12.77	-10.95
200	128.4	131.3	14.03	-12.23
210	133.4	137.7	15.34	-13.58

T / K	C^{o}_{pm} / J·K^{-1}·mol^{-1}	S^{o}_{m} / J·K^{-1}·mol^{-1}	$\Delta_0^T H^{o}_{m}$ / kJ·mol^{-1}	$\Delta_0^T G^{o}_{m}$ / kJ·mol^{-1}
220	138.5	144.0	16.70	-14.99
230	143.7	150.3	18.11	-16.46
240	148.9	156.5	19.58	-17.99
250	154.2	162.7	21.09	-19.59
260	159.5	168.9	22.66	-21.25
270	164.8	175.0	24.28	-22.97
273.15	166.5	176.9	24.80	-23.52
280	170.2	181.1	25.96	-24.75
290	175.5	187.2	27.68	-26.59
298.15	179.9	192.1	29.13	-28.13
300	180.9	193.2	29.47	-28.49
310	186.3	199.2	31.30	-30.45
320	191.6	205.2	33.19	-32.48
330	196.9	211.2	35.13	-34.56
340	202.2	217.1	37.13	-36.70
350	207.4	223.1	39.18	-38.90
360	212.5	229.0	41.28	-41.16
370	217.5	234.9	43.43	-43.48
380	222.5	240.8	45.63	-45.86
390	227.3	246.6	47.88	-48.29
400	232.0	252.4	50.17	-50.79
410	236.5	258.2	52.51	-53.34
420	240.8	263.9	54.90	-55.95
430	245.0	269.7	57.33	-58.62

[a] Crystal structure deposited in the Cambridge Structural Database with refcode LHISTD10 (see the section 3.2 and Table 3 in the main article).

[b] The combined expanded uncertainty of heat capacity $U_c(C_{pm})$ as well as of all calculated thermodynamic values (with 0.95 level of confidence, k=2) is: $U_c(X)$=0.1 X below 10 K; $U_c(X)$=0.03 X in temperature range (10 to 40) K; $U_c(X)$=0.02 X in temperature range (40 to 260) K; $U_c(X)$=0.01 X in temperature range (260 to 350) K; $U_c(X)$=0.02 X in temperature range (350 to 430) K, where X represents the heat capacity or the thermodynamic property. Values are reported with one digit more than is justified by the experimental uncertainty to avoid round-off errors in calculations based on these results.

[c] Extrapolated values.

Table S8. Standard thermodynamic functions of L-phenylalanine in the crystal state[a] at $p = 0.1$ MPa.[b]

T / K	C^{o}_{pm} / J·K^{-1}·mol^{-1}	S^{o}_{m} / J·K^{-1}·mol^{-1}	$\Delta_0^T H^{o}_{m}$ / kJ·mol^{-1}	$\Delta_0^T G^{o}_{m}$ / kJ·mol^{-1}
1[c]	4.412E-03	1.490E-03	1.115E-06	-3.756E-07
2[c]	3.346E-02	1.146E-02	1.709E-05	-5.822E-06
3[c]	1.069E-01	3.714E-02	8.288E-05	-2.854E-05
4[c]	2.397E-01	8.452E-02	2.507E-04	-8.735E-05
5[c]	4.424E-01	1.584E-01	5.856E-04	-2.064E-04
6[c]	7.220E-01	2.626E-01	1.161E-03	-4.143E-04
7[c]	1.082	3.999E-01	2.056E-03	-7.426E-04
8[c]	1.524	5.723E-01	3.353E-03	-1.226E-03
9[c]	2.046	7.812E-01	5.131E-03	-1.899E-03
10[c]	2.647	1.027	7.471E-03	-2.800E-03
11	3.321	1.311	1.045E-02	-3.966E-03
12	4.064	1.631	1.414E-02	-5.434E-03
13	4.871	1.988	1.860E-02	-7.240E-03
14	5.736	2.380	2.390E-02	-9.421E-03
15	6.652	2.807	3.009E-02	-1.201E-02
16	7.613	3.266	3.722E-02	-1.505E-02
17	8.615	3.758	4.533E-02	-1.856E-02
18	9.650	4.279	5.446E-02	-2.257E-02
19	10.71	4.830	6.464E-02	-2.712E-02
20	11.80	5.407	7.589E-02	-3.224E-02
25	17.46	8.643	1.489E-01	-6.714E-02
30	23.21	12.34	2.506E-01	-1.194E-01
35	28.88	16.34	3.809E-01	-1.910E-01
40	34.33	20.56	5.390E-01	-2.832E-01
45	39.49	24.90	7.237E-01	-3.968E-01
50	44.36	29.31	9.334E-01	-5.323E-01
55	48.91	33.76	1.167	-6.900E-01
60	53.18	38.20	1.422	-8.699E-01
65	57.19	42.62	1.698	-1.072
70	60.96	46.99	1.994	-1.296
75	64.54	51.32	2.307	-1.542
80	67.95	55.60	2.639	-1.809
85	71.22	59.82	2.987	-2.098
90	74.38	63.98	3.351	-2.407
95	77.47	68.08	3.730	-2.737
100	80.50	72.13	4.125	-3.088
110	86.50	80.09	4.960	-3.849
120	92.44	87.87	5.855	-4.689
130	98.36	95.50	6.809	-5.606
140	104.3	103.0	7.822	-6.599
150	110.2	110.4	8.895	-7.666
160	116.2	117.7	10.03	-8.806
170	122.2	124.9	11.22	-10.02
180	128.2	132.1	12.47	-11.31
190	134.3	139.2	13.78	-12.66
200	140.3	146.2	15.16	-14.09
210	146.5	153.2	16.59	-15.59

T / K	$C^{\mathrm{o}}_{p\mathrm{m}}$ / J·K^{-1}·mol^{-1}	$S^{\mathrm{o}}_{\mathrm{m}}$ / J·K^{-1}·mol^{-1}	$\Delta_0^T H^{\mathrm{o}}_{\mathrm{m}}$ / kJ·mol^{-1}	$\Delta_0^T G^{\mathrm{o}}_{\mathrm{m}}$ / kJ·mol^{-1}
220	152.6	160.2	18.08	-17.15
230	158.8	167.1	19.64	-18.79
240	165.0	174.0	21.26	-20.49
250	171.2	180.8	22.94	-22.27
260	177.5	187.7	24.68	-24.11
270	183.8	194.5	26.49	-26.02
273.15	185.8	196.6	27.07	-26.64
280	190.2	201.3	28.36	-28.00
290	196.5	208.1	30.29	-30.05
298.15	201.7	213.6	31.92	-31.77
300	202.9	214.8	32.29	-32.16
310	209.3	221.6	34.35	-34.34
320	215.8	228.4	36.48	-36.59
330	222.2	235.1	38.67	-38.91
340	228.6	241.8	40.92	-41.30
350	235.0	248.5	43.24	-43.75
360	241.4	255.2	45.62	-46.27
370	247.8	261.9	48.07	-48.85
380	254.1	268.6	50.58	-51.50
390	260.4	275.3	53.15	-54.22
400	266.6	282.0	55.79	-57.01
410	272.8	288.7	58.48	-59.86
420	278.8	295.3	61.24	-62.78

[a] Crystal structure deposited in the Cambridge Structural Database with refcode QQQAUJ05 (see the section 3.2 and Table 3 in the main article).

[b] The combined expanded uncertainty of heat capacity $U_c(C_{p\mathrm{m}})$ as well as of all calculated thermodynamic values (with 0.95 level of confidence, k=2) is: $U_c(X)$=0.1 X below 10 K; $U_c(X)$=0.03 X in temperature range (10 to 40) K; $U_c(X)$=0.02 X in temperature range (40 to 260) K; $U_c(X)$=0.01 X in temperature range (260 to 350) K; $U_c(X)$=0.02 X in temperature range (350 to 430) K, where X represents the heat capacity or the thermodynamic property. Values are reported with one digit more than is justified by the experimental uncertainty to avoid round-off errors in calculations based on these results.

[c] Extrapolated values.

Table S9. Standard thermodynamic functions of L-proline in the crystal state[a] at $p = 0.1$ MPa.[b]

T / K	C^{o}_{pm} / J·K^{-1}·mol^{-1}	S^{o}_{m} / J·K^{-1}·mol^{-1}	$\Delta_0^T H^{o}_{m}$ / kJ·mol^{-1}	$\Delta_0^T G^{o}_{m}$ / kJ·mol^{-1}
1[c]	1.008E-03	3.311E-04	2.490E-07	-8.205E-08
2[c]	8.499E-03	2.760E-03	4.163E-06	-1.358E-06
3[c]	3.006E-02	9.673E-03	2.193E-05	-7.094E-06
4[c]	7.417E-02	2.372E-02	7.179E-05	-2.308E-05
5[c]	1.499E-01	4.773E-02	1.808E-04	-5.785E-05
6[c]	2.662E-01	8.465E-02	3.851E-04	-1.228E-04
7[c]	4.318E-01	1.374E-01	7.296E-04	-2.324E-04
8[c]	6.543E-01	2.090E-01	1.268E-03	-4.040E-04
9[c]	9.401E-01	3.019E-01	2.059E-03	-6.575E-04
10[c]	1.294	4.186E-01	3.171E-03	-1.016E-03
11	1.718	5.613E-01	4.671E-03	-1.503E-03
12	2.214	7.315E-01	6.631E-03	-2.147E-03
13	2.780	9.306E-01	9.122E-03	-2.976E-03
14	3.413	1.159	1.221E-02	-4.018E-03
15	4.108	1.418	1.597E-02	-5.305E-03
16	4.860	1.707	2.045E-02	-6.865E-03
17	5.663	2.026	2.571E-02	-8.729E-03
18	6.508	2.373	3.179E-02	-1.093E-02
19	7.388	2.748	3.873E-02	-1.348E-02
20	8.297	3.150	4.657E-02	-1.643E-02
25	13.06	5.509	9.987E-02	-3.786E-02
30	17.85	8.314	1.772E-01	-7.226E-02
35	22.58	11.42	2.783E-01	-1.215E-01
40	27.15	14.74	4.027E-01	-1.868E-01
45	31.47	18.19	5.493E-01	-2.691E-01
50	35.50	21.71	7.169E-01	-3.688E-01
55	39.22	25.27	9.038E-01	-4.863E-01
60	42.65	28.84	1.109	-6.216E-01
65	45.82	32.38	1.330	-7.746E-01
70	48.74	35.88	1.566	-9.453E-01
75	51.46	39.34	1.817	-1.133
80	54.02	42.74	2.081	-1.339
85	56.44	46.09	2.357	-1.561
90	58.77	49.38	2.645	-1.799
95	61.02	52.62	2.945	-2.054
100	63.24	55.81	3.255	-2.325
110	67.65	62.04	3.910	-2.915
120	72.04	68.11	4.608	-3.566
130	76.44	74.05	5.350	-4.277
140	80.84	79.88	6.137	-5.046
150	85.26	85.61	6.967	-5.874
160	89.68	91.25	7.842	-6.758
170	94.11	96.82	8.761	-7.699
180	98.53	102.3	9.724	-8.695
190	102.9	107.8	10.73	-9.745
200	107.3	113.2	11.78	-10.85
210	111.6	118.5	12.88	-12.01

T / K	C°_{pm} / J·K^{-1}·mol^{-1}	S°_{m} / J·K^{-1}·mol^{-1}	$\Delta_0^T H^{\circ}_{m}$ / kJ·mol^{-1}	$\Delta_0^T G^{\circ}_{m}$ / kJ·mol^{-1}
220	116.0	123.8	14.02	-13.22
230	120.2	129.0	15.20	-14.48
240	124.5	134.3	16.42	-15.80
250	128.8	139.4	17.69	-17.17
260	133.2	144.6	19.00	-18.59
270	137.5	149.7	20.35	-20.06
273.15	138.9	151.3	20.79	-20.53
280	141.9	154.8	21.75	-21.58
290	146.4	159.8	23.19	-23.16
298.15	150.1	163.9	24.40	-24.47
300	150.9	164.8	24.68	-24.78
310	155.6	169.9	26.21	-26.45
320	160.3	174.9	27.79	-28.18
330	165.1	179.9	29.41	-29.95
340	170.1	184.9	31.09	-31.77
350	175.1	189.9	32.82	-33.65
360	180.3	194.9	34.59	-35.57
370	185.6	199.9	36.42	-37.55
380	190.7	204.9	38.30	-39.57
390	195.6	209.9	40.24	-41.64
400	200.1	215.0	42.21	-43.77
410	204.1	219.9	44.24	-45.94
420	207.5	224.9	46.29	-48.17
430	210.2	229.8	48.38	-50.44

[a] Crystal structure deposited in the Cambridge Structural Database with refcode PROLIN (see the section 3.2 and Table 3 in the main article).

[b] The combined expanded uncertainty of heat capacity $U_c(C_{pm})$ as well as of all calculated thermodynamic values (with 0.95 level of confidence, k=2) is: $U_c(X)$=0.1 X below 10 K; $U_c(X)$=0.03 X in temperature range (10 to 40) K; $U_c(X)$=0.02 X in temperature range (40 to 260) K; $U_c(X)$=0.01 X in temperature range (260 to 350) K; $U_c(X)$=0.02 X in temperature range (350 to 430) K, where X represents the heat capacity or the thermodynamic property. Values are reported with one digit more than is justified by the experimental uncertainty to avoid round-off errors in calculations based on these results.

[c] Extrapolated values.

Table S10. Standard thermodynamic functions of L-tryptophan in the crystal state[a] at p = 0.1 MPa.[b]

T / K	C^{o}_{pm} / J·K^{-1}·mol^{-1}	S^{o}_{m} / J·K^{-1}·mol^{-1}	$\Delta_0^T H^{o}_{m}$ / kJ·mol^{-1}	$\Delta_0^T G^{o}_{m}$ / kJ·mol^{-1}
1[c]	1.017E-02	3.478E-03	2.595E-06	-8.829E-07
2[c]	7.366E-02	2.582E-02	3.834E-05	-1.331E-05
3[c]	2.255E-01	8.101E-02	1.795E-04	-6.353E-05
4[c]	4.856E-01	1.788E-01	5.255E-04	-1.896E-04
5[c]	8.634E-01	3.255E-01	1.190E-03	-4.374E-04
6[c]	1.361	5.251E-01	2.292E-03	-8.582E-04
7[c]	1.974	7.795E-01	3.950E-03	-1.506E-03
8[c]	2.697	1.089	6.277E-03	-2.436E-03
9[c]	3.521	1.453	9.379E-03	-3.702E-03
10[c]	4.436	1.871	1.335E-02	-5.360E-03
11[c]	5.431	2.340	1.828E-02	-7.461E-03
12	6.496	2.858	2.424E-02	-1.006E-02
13	7.621	3.422	3.129E-02	-1.319E-02
14	8.796	4.029	3.949E-02	-1.691E-02
15	10.01	4.677	4.889E-02	-2.126E-02
16	11.26	5.363	5.953E-02	-2.628E-02
17	12.54	6.084	7.143E-02	-3.200E-02
18	13.83	6.837	8.461E-02	-3.846E-02
19	15.14	7.620	9.909E-02	-4.569E-02
20	16.46	8.430	1.149E-01	-5.371E-02
25	23.02	12.81	2.136E-01	-1.066E-01
30	29.33	17.57	3.446E-01	-1.824E-01
35	35.26	22.54	5.063E-01	-2.826E-01
40	40.81	27.61	6.966E-01	-4.080E-01
45	46.06	32.73	9.139E-01	-5.589E-01
50	51.04	37.84	1.157	-7.353E-01
55	55.78	42.93	1.424	-9.372E-01
60	60.31	47.98	1.714	-1.165
65	64.65	52.98	2.027	-1.417
70	68.84	57.92	2.360	-1.694
75	72.89	62.81	2.715	-1.996
80	76.82	67.64	3.089	-2.322
85	80.67	72.41	3.483	-2.672
90	84.43	77.13	3.896	-3.046
95	88.14	81.80	4.327	-3.444
100	91.80	86.41	4.777	-3.864
110	99.01	95.50	5.731	-4.774
120	106.2	104.4	6.757	-5.774
130	113.2	113.2	7.854	-6.862
140	120.3	121.9	9.022	-8.037
150	127.4	130.4	10.26	-9.298
160	134.5	138.8	11.57	-10.65
170	141.7	147.2	12.95	-12.08
180	148.9	155.5	14.40	-13.59
190	156.2	163.8	15.93	-15.19
200	163.5	172.0	17.53	-16.86
210	170.9	180.1	19.20	-18.62

T / K	C^{o}_{pm} / J·K^{-1}·mol^{-1}	S^{o}_{m} / J·K^{-1}·mol^{-1}	$\Delta_0^T H^{o}_{m}$ / kJ·mol^{-1}	$\Delta_0^T G^{o}_{m}$ / kJ·mol^{-1}
220	178.3	188.2	20.95	-20.47
230	185.8	196.3	22.77	-22.39
240	193.3	204.4	24.66	-24.39
250	200.9	212.4	26.63	-26.48
260	208.5	220.5	28.68	-28.64
270	216.1	228.5	30.80	-30.89
273.15	218.5	231.0	31.49	-31.61
280	223.7	236.5	33.00	-33.21
290	231.4	244.5	35.28	-35.61
298.15	237.6	250.9	37.19	-37.63
300	239.0	252.4	37.63	-38.10
310	246.6	260.4	40.06	-40.66
320	254.1	268.3	42.56	-43.31
330	261.6	276.3	45.14	-46.03
340	269.1	284.2	47.79	-48.83
350	276.4	292.1	50.52	-51.71
360	283.6	300.0	53.32	-54.67
370	290.7	307.8	56.19	-57.71
380	297.6	315.7	59.13	-60.83
390	304.3	323.5	62.14	-64.03
400	310.9	331.3	65.22	-67.30
410	317.2	339.1	68.36	-70.65
420	323.3	346.8	71.56	-74.08
430	329.1	354.4	74.82	-77.59

[a] Crystal structure deposited in the Cambridge Structural Database with refcode VIXQOK (see the section 3.2 and Table 3 in the main article).

[b] The combined expanded uncertainty of heat capacity $U_c(C_{pm})$ as well as of all calculated thermodynamic values (with 0.95 level of confidence, k=2) is: $U_c(X)$=0.1 X below 10 K; $U_c(X)$=0.03 X in temperature range (10 to 40) K; $U_c(X)$=0.02 X in temperature range (40 to 260) K; $U_c(X)$=0.01 X in temperature range (260 to 350) K; $U_c(X)$=0.02 X in temperature range (350 to 430) K, where X represents the heat capacity or the thermodynamic property. Values are reported with one digit more than is justified by the experimental uncertainty to avoid round-off errors in calculations based on these results.

[c] Extrapolated values.

Table S11. Standard thermodynamic functions of L-tyrosine in the crystal state[a] at $p = 0.1$ MPa.[b]

T / K	C^{o}_{pm} / J·K^{-1}·mol^{-1}	S^{o}_{m} / J·K^{-1}·mol^{-1}	$\Delta^{T}_{0}H^{o}_{m}$ / kJ·mol^{-1}	$\Delta^{T}_{0}G^{o}_{m}$ / kJ·mol^{-1}
1[c]	2.372E-03	8.070E-04	6.028E-07	-2.043E-07
2[c]	1.758E-02	6.090E-03	9.063E-06	-3.117E-06
3[c]	5.530E-02	1.946E-02	4.330E-05	-1.509E-05
4[c]	1.228E-01	4.383E-02	1.296E-04	-4.571E-05
5[c]	2.256E-01	8.157E-02	3.007E-04	-1.072E-04
6[c]	3.684E-01	1.347E-01	5.942E-04	-2.140E-04
7[c]	5.548E-01	2.049E-01	1.052E-03	-3.823E-04
8[c]	7.879E-01	2.937E-01	1.719E-03	-6.300E-04
9[c]	1.070	4.023E-01	2.644E-03	-9.763E-04
10[c]	1.404	5.319E-01	3.877E-03	-1.442E-03
11	1.792	6.835E-01	5.471E-03	-2.047E-03
12	2.234	8.580E-01	7.479E-03	-2.816E-03
13	2.732	1.056	9.958E-03	-3.771E-03
14	3.286	1.278	1.296E-02	-4.936E-03
15	3.896	1.526	1.655E-02	-6.336E-03
16	4.561	1.798	2.077E-02	-7.996E-03
17	5.280	2.096	2.569E-02	-9.941E-03
18	6.050	2.419	3.135E-02	-1.220E-02
19	6.870	2.768	3.780E-02	-1.479E-02
20	7.734	3.142	4.510E-02	-1.774E-02
25	12.60	5.376	9.561E-02	-3.879E-02
30	18.09	8.152	1.722E-01	-7.240E-02
35	23.83	11.37	2.769E-01	-1.211E-01
40	29.57	14.93	4.104E-01	-1.867E-01
45	35.15	18.73	5.723E-01	-2.707E-01
50	40.50	22.72	7.615E-01	-3.743E-01
55	45.60	26.82	9.768E-01	-4.981E-01
60	50.44	30.99	1.217	-6.426E-01
65	55.03	35.22	1.481	-8.081E-01
70	59.37	39.45	1.767	-9.948E-01
75	63.49	43.69	2.074	-1.203
80	67.41	47.91	2.402	-1.432
85	71.17	52.11	2.748	-1.682
90	74.80	56.29	3.113	-1.953
95	78.33	60.42	3.496	-2.245
100	81.79	64.53	3.896	-2.557
110	88.64	72.65	4.748	-3.243
120	95.41	80.65	5.669	-4.010
130	102.1	88.56	6.657	-4.856
140	108.9	96.37	7.712	-5.780
150	115.6	104.1	8.834	-6.783
160	122.3	111.8	10.02	-7.862
170	129.0	119.4	11.28	-9.018
180	135.7	127.0	12.60	-10.25
190	142.4	134.5	13.99	-11.56
200	149.1	142.0	15.45	-12.94
210	155.8	149.4	16.98	-14.40

T / K	C^{o}_{pm} / J·K^{-1}·mol^{-1}	S^{o}_{m} / J·K^{-1}·mol^{-1}	$\Delta_0^T H^{o}_{m}$ / kJ·mol^{-1}	$\Delta_0^T G^{o}_{m}$ / kJ·mol^{-1}
220	162.5	156.8	18.57	-15.93
230	169.2	164.2	20.23	-17.53
240	176.0	171.5	21.95	-19.21
250	182.7	178.8	23.75	-20.96
260	189.4	186.1	25.61	-22.79
270	196.2	193.4	27.53	-24.68
273.15	198.4	195.7	28.16	-25.30
280	203.0	200.7	29.53	-26.66
290	209.9	207.9	31.59	-28.70
298.15	215.5	213.8	33.33	-30.42
300	216.8	215.1	33.73	-30.81
310	223.7	222.4	35.93	-33.00
320	230.6	229.6	38.20	-35.26
330	237.6	236.8	40.54	-37.59
340	244.6	244.0	42.95	-40.00
350	251.7	251.2	45.43	-42.47
360	258.8	258.4	47.99	-45.02
370	265.9	265.5	50.61	-47.64
380	273.1	272.7	53.31	-50.33
390	280.3	279.9	56.07	-53.09
400	287.5	287.1	58.91	-55.93
410	294.7	294.3	61.82	-58.83
420	302.0	301.5	64.81	-61.81
430	309.3	308.7	67.86	-64.86

[a] Crystal structure deposited in the Cambridge Structural Database with refcode LTYROS11 (see the section 3.2 and Table 3 in the main article).

[b] The combined expanded uncertainty of heat capacity $U_c(C_{pm})$ as well as of all calculated thermodynamic values (with 0.95 level of confidence, k=2) is: $U_c(X)$=0.1 X below 10 K; $U_c(X)$=0.03 X in temperature range (10 to 40) K; $U_c(X)$=0.02 X in temperature range (40 to 260) K; $U_c(X)$=0.01 X in temperature range (260 to 350) K; $U_c(X)$=0.02 X in temperature range (350 to 430) K, where X represents the heat capacity or the thermodynamic property. Values are reported with one digit more than is justified by the experimental uncertainty to avoid round-off errors in calculations based on these results.

[c] Extrapolated values.
